# Preventive Effects of Folic Acid Supplementation on Adverse Maternal and Fetal Outcomes

**DOI:** 10.1371/journal.pone.0097273

**Published:** 2014-05-19

**Authors:** Min Woo Kim, Ki Hoon Ahn, Ki-Jin Ryu, Soon-Cheol Hong, Ji Sung Lee, Alejandro A. Nava-Ocampo, Min-Jeong Oh, Hai-Joong Kim

**Affiliations:** 1 Department of Obstetrics and Gynecology, Korea University College of Medicine, Seoul, South Korea; 2 Biostatistical Consulting Unit, Soonchunhyang University Medical Center, Seoul, South Korea; 3 Division of Clinical Pharmacology and Toxicology, Hospital for Sick Children, Toronto, Ontario, Canada; 4 Pharmacological Research and Applied Solutions–PharmaReasons, Toronto, Ontario, Canada; Tabriz University of Medical Sciences, Iran (Republic of Islamic)

## Abstract

Although there is accumulating evidence regarding the additional protective effect of folic acid against adverse pregnancy outcomes other than neural tube defects, these effects have not been elucidated in detail. We evaluated whether folic acid supplementation is associated with favorable maternal and fetal outcomes. This was a secondary analysis of 215 pregnant women who were enrolled in our prior study. With additional data from telephone interviews regarding prenatal folic acid supplementation, existing demographic, maternal and fetal data were statistically analyzed. The concentration of folic acid in maternal blood was significantly higher following folic acid supplementation (24.6 ng/mL vs.11.8 ng/mL). In contrast, homocysteine level in maternal blood decreased with folic acid supplementation (5.5 µmol/mL vs. 6.8 µmol/mL). The rates of both preeclampsia (odds ratio [OR], 0.27; 95% confidence interval [CI], 0.09–0.76) and small for gestational age (SGA; 9.2% vs. 20.0%; OR, 0.42; 95% CI, 0.18–0.99) were lower in the folic acid supplementation group than those in the control group. Other pregnancy outcomes had no association with folic acid supplementation. The findings indicate that folic acid supplementation may help to prevent preeclampsia and SGA. Further studies are warranted to elucidate the favorable effects of folic acid supplementation on pregnancy outcomes.

## Introduction

Overwhelming evidence supports the use of folic acid supplementation during pregnancy to prevent neural tube defects [1]. Prenatal intake of folic acid has also been associated with better long-term neurodevelopment in offspring [2]. However, whether the protective effects of folic acid extend to other pregnancy outcomes have not been clearly identified [3]. In a population-based prospective cohort study, maternal hyperhomocysteinemia was linked to a higher risk of adiposity and type 2 diabetes in mothers [4] and their offspring [5].

Our prior study showed that lower folate and higher homocysteine concentrations in maternal serum at the time of delivery are associated with unfavorable pregnancy outcomes such as pre-term birth and pre-eclampsia [6]. In this study, we determined whether antenatal folic acid supplementation is associated with favorable maternal and fetal outcomes.

## Materials and Methods

This was a retrospective secondary analysis to a study group described previously [6]. We obtained the approval of the Institutional Review Board in Korea University Anam Hospital (IRB No: ED12248). This study was exempt from participants’ informed consent because the investigator conducting research of this secondary analysis did not obtain information about research subjects via an interaction with them, nor did the investigator obtain identifiable private information. Briefly, the study population included women with singleton pregnancies who delivered at the Korea University Anam Hospital between June 1, 2009, and June 13, 2010 and provided informed consent. Maternal blood samples were collected during antenatal visits or upon admission to the hospital. Cord blood samples were obtained from the umbilical vein immediately after delivery. Plasma total homocysteine concentration was measured using an automated enzymatic assay and homocysteine methyltransferase and d-amino acid oxidase (Toshiba 200FR-NEO Auto Analyzer; Toshiba Medical Systems Co., Ltd., Tokyo, Japan). Folate level was measured using an iodine-125-based radioimmunoassay (Cobra II 5010; Packard, Meriden, CT, USA). Among a total of 227 pregnant women reviewed initially, 215 cases were included in the study. Continuous variables are expressed as mean ± standard deviation or median (interquartile range [IQR]) values, whereas categorical variables are presented using their absolute values and percentages. Student’s *t*-test or the Wilcoxon rank-sum test was used for continuous variables; and the chi-square test or Fisher’s exact test were used for categorical variables. We performed a multivariable logistic regression analysis to analyze the associations between folic acid supplementation with other confounding factors such as parity, familial monthly income, preeclampsia, somatic classification of newborns. All statistical analyses were performed using SAS ver. 9.3 software (SAS Institute, Cary, NC, USA), and statistical significance was defined as a two-tailed *P*<.05.

## Results

We found no significant differences between the two groups regarding maternal age, body mass index, alcohol drinking history, smoking history, economic status, or occupational status. Median parity was 1 (IQR, 0–1) in the women who did not take a folic acid supplement and 0 (IQR, 0–1) in the supplementation group (Table 1). The concentration of folic acid in maternal blood was significantly higher with folic acid supplementation (24.6 ng/mL [14.4–37.0] vs. 11.8 ng/mL [7.4–18.5]; *P*<.0001). In contrast, homocysteine level in maternal blood decreased with folic acid supplementation (5.5 µmol/mL [3.9–8.0] vs. 6.8 µmol/mL [4.9–9.7]; *P* = .0163). The incidence of preeclampsia was lower in the folic acid supplementation group than that in the control group (4.2% vs. 14.1%; *P* = .0076). With the folic acid supplementation, the rate of small for gestational age was decreased. No differences were observed between the two groups concerning the rates of gestational diabetes, placenta previa, placental abruption, preterm births, or birth weight (Table 2). We performed multivariate logistic regression analysis after adjusting for confounding factors, which included parity, familial monthly income, preeclampsia and prenatal intake of folic acid. Based on these analyses, the risk of preeclampsia decreased with folic acid supplementation (odds ratio [OR], 0.27; 95% confidence interval [CI], 0.09–0.76; *P = *.014; Table 3). The risk of small for gestational age (SGA) was also lowered by folic acid intake (OR = 0.42; 95% CI = 0.18–0.99; *P = *.0047; Tables 4). Higher parity was associated with lower SGA (OR = 0.41; 95% CI = 0.19–0.89; *P = *.0023).

**Table 1:** Demographic and clinical characteristics of the participants.

	Prenatal intake of folic acid		
	Negative (n = 81)	Positive (n = 134)	P value
Age (years)	31.3±4.7	31.9±3.9	0.3588†
Parity, median (IQR)*	1 (0–1)	0 (0–1)	0.0453
BMI	25.1±3.0	24.6±3.2	0.2386
Alcohol history, n (%)			0.1392‡
Drinker	2 (2.4)	0 (0.0)	
Non-drinker	83 (97.7)	142 (100.0)	
Smoking history, n (%)			–
Smoker	–	–	
Non-smoker	85 (100.0)	142 (100.0)	
Family monthly income ($US), n (%)			0.0652¶
<2000	20 (23.5)	24 (16.9)	
2000–5000	63 (74.1)	104 (73.2)	
>5000	2 (2.4)	14 (9.9)	
Occupation, n (%)			0.7095¶
Yes	32 (37.7)	57 (40.1)	
No	53 (62.4)	85 (59.9)	

IQR, interquartile range; BMI, body mass index.

Data are summarized as the mean ± standard deviation or % (n).

*Data are median (IQR). Data were analyzed using the Wilcoxon rank-sum test.

† P value, Student’s *t*-test.

‡ P value, Fisher’s exact test.

¶ P value, chi-square test.

**Table 2:** Maternal and fetal outcomes.

	Prenatal intake of folic acid		
	Negative (n = 81)	Positive (n = 134)	P value
**Maternal outcomes**			
Gestational age (weeks), mean ± SD	36.3±3.9	36.1±3.8	0.9960†
Gestational diabetes, n (%)	4 (4.7)	15 (10.6)	0.1230‡
Preeclampsia, n (%)	12 (14.1)	6 (4.2)	0.0076‡
Placenta previa, n (%)	3 (3.5)	10 (7.0)	0.3801¶
Placenta abruption, n (%)	5 (5.9)	5 (3.5)	0.5072¶
Preterm premature rupture of membranes, n (%)	9 (10.6)	24 (16.9)	0.609‡
**Fetal outcomes**			
Birth weight (kg)	2.7±0.8	2.8±0.8	0.2956†
Preterm birth (<37 weeks)	35 (41.2)	62 (43.7)	0.7141‡
Birth size classification			0.0195‡
SGA (<10 percentile for GA)	17 (20.0)	13 (9.2)	
No SGA	68 (80.0)	129 (90.8)	
**Maternal blood levels**			
Folate levels (ng/mL)*	11.8 (7.4–18.5)	24.6 (14.4–37.0)	<0.0001
Homocysteine levels (µmol/mL)*	6.8 (4.9–9.7)	5.5 (3.9–8.0)	0.0163

AGA, appropriate for gestational age; GA, gestational age; SGA, small for gestational age; LGA, large for gestational age.

Data are summarized as mean ± standard deviation or % (n) values.

**Data are presented as median (IQR) values. Data were analyzed using Wilcoxon rank-sum test.

† P value, Student’s *t*-test.

‡ P value, chi-square test.

¶ P value, Fisher’s exact test.

**Table 3:** Odds ratio (OR) for preeclampsia according to the multivariable logistic regression analysis.

	OR	95% CI	P value
Parity	1.07	0.49–2.31	0.869
Family monthly income ($US)			
<2000	Ref.		
2000–5000	0.93	0.28–3.06	0.9
>5000	1.13	0.11–11.77	0.921
Prenatal intake of folic acid			
Negative	Ref.		
Positive	0.27	0.09–0.76	0.014

Ref., reference; CI, confidence interval.

**Table 4:** Odds ratios (ORs) for the somatic classification of newborns according to the multivariate logistic regression analysis.

	SGA vs. No SGA		
	OR	95% CI	P-value
Parity	0.41	0.19–0.89	0.023
Family monthly income ($US)			
<2000	Ref.		
2000–5000	1.40	0.47–4.20	0.546
>5000	1.34	0.21–8.73	0.76
Preeclampsia			
No	Ref.		
Yes	6.18	2.04–18.73	0.001
Prenatal intake of folic acid			
Negative	Ref.		
Positive	0.42	0.18–0.99	0.047

SGA, small for gestational age; Ref., reference; CI, confidence interval.

## Discussion

In our study, folic acid supplementation decreased the risk of small for gestational age. It is known that folic acid plays a role in both placental development and fetal growth, as it contributes to protein, lipid, and DNA synthesis. Folate contains a methyl group, and it can affect DNA methylation by altering 1-carbon metabolism. The epigenetic regulation of growth processes through folic acid supplementation may contribute to embryonic development and fetal growth [7]. In this theoretical framework, folic acid has been reported to be positively related to intrauterine fetal growth [8]–[10]. In a population-based prospective study, the Generation R Study, birth weight was 68 g higher in women who started folic acid supplementation preconceptionally and 53 g higher in those who started after pregnancy recognition compared with the birth weight for mothers who did not take folic acid supplements [11]. In contrast, homocysteine has been reported to be inversely related to placental and fetal growth [12]. Considering that the homocysteine level was lower in the folic acid supplementation group, the association between folic acid level and birth weight would be affected directly or indirectly by the folate-homocysteine pathway.

In our study, the concentration of folic acid was elevated and the homocysteine level decreased significantly in women with history of folic acid supplementation. These findings inform that the study pregnant women followed their physician’s recommendations well, so they should be encouraged to consume folic acid or informed about its beneficial role. It is well known that fewer than 20% of women comply with folic acid supplementation during early pregnancy, and more than half of pregnancies are unintended or unplanned [13], [14]. Moreover, there is variability in the recommended amount and duration of folic acid supplementation between countries and clinicians. Under this circumstance, the necessity of mandatory folate fortification or consensus guideline is critical [15], [16]. In our study, according to the folic acid supplementation policy of our hospital (women in good health status consume a multivitamin containing folic acid (0.4–1.0 mg) daily for at least 2–3 months before conception and throughout pregnancy and the postpartum period), most women in the supplementary group were taking folic acid at the time of blood sampling. Considering the additional protective effect of folic acid against adverse pregnancy outcomes, folic acid supplementation should be recommended throughout pregnancy.

The relationship between folic acid and various pregnancy outcomes such as preeclampsia, placental abruption, abortion, preterm birth, and stillbirth has been investigated [17]–[20], however, the results are still controversial. Some studies have demonstrated that folic acid prevents adverse pregnancy outcomes, whereas others have failed to confirm these favorable effects [3], [21]–[23]. Regarding the preterm birth, we demonstrated in our previous study the relationship between folate concentrations and preterm delivery [6], however in the current secondary analysis of that study we failed to elucidate an association between folic acid supplementation and the rate of preterm birth.

The largest study to report the protective effect of folic acid supplementation against preterm birth was conducted by Bukowski et al. [21]. In a secondary analysis of the FASTER trial, they described that preconceptional folic acid supplementation for 1 year or longer is associated with a 70% decrease in the risk of spontaneous preterm delivery at 20–28 weeks and a 50% decrease in the risk of spontaneous preterm delivery at 28–32 weeks compared with no supplementation. The differing results between that study and our study are believed to be due to the rate of preterm birth in the study population. They reported that 2660 women (7.7%) delivered prematurely before 37 weeks, and spontaneous preterm births before 37 weeks were reported for 1658 women (4.8%). Conversely, the rates of preterm birth in our study were 41.2% in the group without supplementation and 43.7% in the folic acid supplementation group. This high incidence of preterm birth is attributed to the fact that the patient population in our institution primarily consisted of high-risk patients who transferred from other local hospitals. Thus, the high-risk status of the patients might have diluted the effect of folic acid supplementation. In contrast, as we previously reported an association between folic acid concentration and the rate of preterm birth, this difference may also have arisen from the duration of folic acid supplementation. We did not consider the duration of supplementation, and this is a limitation of our study.

Accumulating evidence indicates the role of folic acid in fetal development and placentation [24], [25]. Folic acid plays a physiological role in cell proliferation and DNA replication. If folic acid concentration is low in maternal blood, plasma homocysteine levels are elevated as a consequence. Folic acid may be involved in trophoblastic invasion during the early stage of placental development [26]. Homocysteine, a metabolite of the amino acid methionine, is an independent risk factor for cardiovascular disease because of its adverse effects on endothelial function [27], [28]. Because preeclampsia is regarded as a systematic vascular disease arising from maternal endothelial dysfunction, hyperhomocysteinemia may contribute to the incidence of preeclampsia [29]. In this context, folic acid supplementation could decrease the prevalence of preeclampsia, as demonstrated in many studies, including ours [6], [30]–[32]. In particular, considering the vegetable-rich diet of the Korean population, this finding caused us to reevaluate the merits of folic acid supplementation. It was recently suggested that hyperhomocysteinemia alone does not sufficiently cause preeclampsia. In an animal study, no increase in the incidence of hypertension or proteinuria was observed in methylenetetrahydrofolate reductase-deficient mice. However, a low-folate diet was associated with proteinuria and growth restriction. These findings may highlight, along with our result, the importance of folic acid on pregnancy outcomes [33]. Lastly, our result showed that higher parity is associated with lower SGA. It is consistent with the results of other studies where primiparity is an independent risk factor for SGA [34], [35].

Concerning the study’s limitations, this was an observational study conducted in a tertiary care hospital; thus, the findings may not be representative of the general population. Nonetheless, our data from reliable statistical analyses are clinically meaningful to present information about the association between folic acid supplementation and various pregnancy outcomes.

The data indicate that folic acid supplementation may be helpful in reducing the incidence of preeclampsia and SGA. To clarify the optimal amount and duration of folic acid supplementation for improving pregnancy outcomes, further studies are warranted.
